# On the Occasional Organic Union of Contiguous Teeth

**Published:** 1852-07

**Authors:** S. J. A. Salter


### ANATOMY AND PHYSIOLOGY.

*On the Occasional Organic Union of Contiguous Teeth.* By S. J. A. SALTER, M. B., etc. (Communicated by THOMAS BELL, Esq., Sec. R. S.)—The object of this paper is to enumerate fresh examples of the so-called "bony" union of contiguous teeth; to explain the true nature of that union, as exhibited by microscopical scrutiny; and to suggest the probable mode of its formation.

1. The fresh instances here recorded are, a united dens sapientiæ and supernumerary tooth of the lower jaw; a cuspidatus and lateral incisor of the lower jaw united; and a specimen exhibiting the lateral and central incisors of the lower jaw in the same condition. These are particularly described.

2. In the next place, the nature of the uniting medium is shown to be dentine and not crusta petrosa, (as stated previously by writers on this subject.) This is demonstrated by microscopical drawings of sections of the teeth.

3. The probable mode by which this abnormity is produced is explained thus:—The pulps of the teeth form in their usual place and usual relation, with this one exception, that the portion of the capsule which should separate them is wanting. The result is, that they come in contact, and when the soft dentinal elements commence calcifying they unite, and the tubes of the two teeth become continuous. There is no enamel between the teeth where the crowns are in contact. The author further remarks, that all the teeth, and in both jaws, have been subjects of this peculiar condition. The paper concludes with a few practical remarks—that, in the majority of cases, the union could not be discovered while the teeth were in the mouth; and that, even where it could be discovered, both teeth must necessarily be removed together, should extraction of either be required, as there appears no means of separating them.

Mr. Streeter would ask whether these teeth might not be examples of duplex formation, like the double uterus, etc., and not the result of union. The inscription on the drawing sent round, certainly describes the teeth as the bicuspid and the incisor, but they looked like a duplex formation, and he did not see why that process should not occur in the teeth.

A Visiter thought, that if such were the case, the regular number of the teeth would be imperfect, which did not in this instance appear to be the case.—*Trans. Royal Med. and Chir. Society in London Medical Times.*

